# Preoperative albumin–bilirubin score as a prognostic indicator in patients with stage III colon cancer

**DOI:** 10.1038/s41598-022-19329-8

**Published:** 2022-09-01

**Authors:** Hyun Gu Lee, Seok-Byung Lim, Jong Lyul Lee, Chan Wook Kim, Yong Sik Yoon, In Ja Park, Jin Cheon Kim

**Affiliations:** Division of Colon and Rectal Surgery, Department of Surgery, Asan Medical Center, University of Ulsan College of Medicine, 88, Olympic-Ro 43-Gil, Songpa-Gu, Seoul, 05505 Korea

**Keywords:** Cancer, Oncology

## Abstract

The albumin–bilirubin (ALBI) score was created to assess the severity of liver dysfunction and to predict prognosis of hepatocellular carcinoma. Purpose of this study was to investigate the prognostic value of the ALBI score in patients with stage III colon cancer using propensity score matching (PSM) analysis. This study analyzed 510 patients with stage III colon cancer who had surgery between 2014 and 2015. The ALBI score was calculated as follows: (log_10_ bilirubin (μmol/L) $\times$ 0.66) + (albumin (g/L) $\times$ −0.0852), and the optimal cut-off value was determined using a receiver operating characteristic analysis and the Youden Index. According to the calculated cut-off value, patients were divided into two groups: Group A (ALBI ≤  − 2.54) and Group B (ALBI >  − 2.54). The average ALBI score was − 2.68 (from − 3.39 to − 0.69). Group A had a significantly higher 5-year disease-free survival rate (85.5% vs 75.7%, *p* = 0.02), 5-year cancer-specific survival rate (93.7% vs 84.4%, p = 0.02), and 5-year overall survival rate (90.6% vs 77.4%, *p* = 0.01) than Group B. High ALBI scores were found to be an independent risk factor for both disease-free survival (HR 1.68, *p* = 0.048) and cancer-specific survival (HR 2.24, *p* = 0.028). The preoperative ALBI score was found to be a promising prognostic indicator for predicting recurrence and survival in patients with stage III colon cancer in this study. Because the ALBI score is simple and inexpensive to obtain, it has the potential to be a useful clinical marker for colon cancer patients.

## Introduction

Despite advances in early detection, surgical techniques, and adjuvant treatment strategies, colorectal cancer remains the third leading cause of cancer-related deaths worldwide^1^. The tumor-nodes-metastasis system has long been used to predict the prognosis of colorectal cancer patients. However, the prognosis of patients with the same stage can vary, and numerous studies have been conducted to identify other prognostic markers that can predict recurrence and survival in clinical practice easily, accurately, and at a low cost^2,3^.

Preoperative liver function and pre-existing liver disease have been linked to increased postoperative morbidity and mortality^4–6^ and poor survival outcomes in colon cancer patients^5,7^. The Child–Pugh (C–P) score (which includes serum albumin and bilirubin levels, prothrombin prolongation time, ascites, and hepatic encephalopathy) is a widely used tool for assessing liver function and the severity of chronic liver disease. It does, however, have limitations, such as a hazy grading system for assessing ascites and hepatic encephalopathy, which can be influenced by subjective interpretation^8^.

The albumin–bilirubin (ALBI) score was developed to overcome the limitations of the C–P score for assessing liver function in patients with hepatocellular carcinoma^9^. The ALBI score is much simpler and more objective than the C-P score because it is based on only two variables, serum albumin, and bilirubin. Serum albumin and bilirubin levels can reflect the synthetic and metabolic function of the liver, and serum albumin levels are also a good indicator of the nutritional status of patients. The ALBI score has been shown to be useful in predicting the prognosis of patients with liver cirrhosis and hepatocellular carcinoma^10–12^. Furthermore, several studies found that the preoperative ALBI score was a prognostic factor in patients with gastric cancer and pancreatic cancer^13,14^, implying that the ALBI score could be proposed as a new prognostic indicator of cancers other than hepatocellular carcinoma.

Recent research has linked a high ALBI score to a worse oncologic outcome in metastatic colorectal cancer^15,16^. According to these studies, the functional impact of the hepatic disease remains an important factor in the overall prognosis of metastatic colorectal cancer^15,16^. Another recent study compared postoperative complications and overall survival in colorectal cancer patients treated with radical resection based on the ALBI score^17^. Although this study suggested that the preoperative ALBI score could be a useful prognostic factor in stage III colorectal cancer, it had limitations such as a small number of included patients, and the results were drawn without considering the effect of adjuvant chemotherapy. As a result, the oncologic significance of the ALBI score in stage III colon cancer has yet to be fully assessed. The goal of this study was to investigate if the ALBI score could predict perioperative morbidity and mortality, as well as long-term oncologic outcomes in patients with stage III colon cancer who were treated with radical resection and adjuvant chemotherapy.

## Patients and methods

### Study population

Between January 2014 and December 2015, 3,606 patients underwent surgical resection for colorectal cancer at Asan Medical Center, Seoul, Korea. Of these patients, 510 patients undergoing curative-intent resection for stage III colon adenocarcinoma were included in this study and retrospectively analyzed. Patients with hereditary colon cancer, colon cancer associated with inflammatory bowel disease, a history of cancer, or concurrent other cancer were excluded. Following the 1964 Helsinki Declaration, the study protocol was approved by the institutional review board of Asan Medical Center (Registration number: 2021-1854).

### Clinical parameters and ALBI score

All clinical data were gathered retrospectively from medical records in the hospital’s database. Preoperative blood tests including serum albumin and bilirubin concentrations were performed within 7 days of surgery. The cut-off values of serum albumin and bilirubin levels were set according to the normal reference ranges used at our institutional clinical laboratory. The ALBI score was calculated as follows: (log_10_ bilirubin $\times$ 0.66) + (albumin $\times$ −0.0852), where bilirubin is measured in μmol/L and albumin is measured in g/L^9^. Age at diagnosis was included in the analysis with a cut-off 65 years, which is used as a chronologic definition of an older adult in the NCCN guidelines ‘Older Adult Oncology’. BMI categories were based on Asian criteria^18^ [underweight (< 18.5), normal weight (18.5–23), overweight (23–25) and obese (> 25)]. The upper-limit cut-off value of preoperative serum carcinoembryonic antigen (CEA) level was set at 5 ng/ml according to previous studies^19^ that demonstrated CEA ≥ 5 ng/ml as a prognostic indicator. Postoperative complications were graded based on severity, with severe complications defined as Clavien–Dindo grade IIIb or higher ^20^.

### Surveillance and oncologic outcomes

Patients were monitored regularly according to our institution’s guidelines, which included physical examinations, serum carcinoembryonic antigen (CEA) levels, computed tomography scans, and colonoscopies. The presence of tumor regrowth in the site of anastomosis or the bed of the primary resection was defined as locoregional recurrence, whereas systemic recurrence was defined as the presence of recurrence beyond the surgical fields. Imaging studies and colonoscopy were used to detect recurrences, which were then confirmed histologically. As a measure of survival, the cumulative 5-year disease-free survival (DFS), cancer-specific survival (CSS) and overall survival (OS) rates were calculated.

### Statistical analysis

The optimal cut-off value of the ALBI score was determined using a receiver operating characteristic (ROC) analysis for 5-year CSS and the Youden Index^21^. Patients were divided into two groups based on the calculated cut-off value. Pearson’s chi-squared test or Fisher’s exact test for categorical variables and an independent sample *t* test for continuous variables were used in comparing patient characteristics between the two groups. Propensity score-matching (PSM) using logistic regression analysis was performed to adjust differences in baseline characteristics, including age, sex, BMI, and tumor stage. PSM was one-to-one without replacement using closest propensity scores for the two ALBI score groups. The Kaplan–Meier method and log-rank test were used to analyze survival outcomes. Univariate and multivariate analyses of factors associated with survival outcomes were performed using Cox proportional hazard regression analyses. The default setting for the numerical calculation was from the PSM groups; otherwise, the settings were specifically mentioned. All statistical analyses were performed using SPSS software (version 25.0; IBM Corp, Armonk, NY), and *p*-values < 0.05 were considered statistically important.

### Ethical approval

The study protocol was approved by the Institutional Review Board of Asan Medical Center in accordance with the 1964 Helsinki Declaration.

### Informed consent

The review board waived the requirement of informed consent, as this study was a retrospective analysis.

## Results

### Patient characteristics and determination of risk groups according to ALBI score

**Figure 1:**
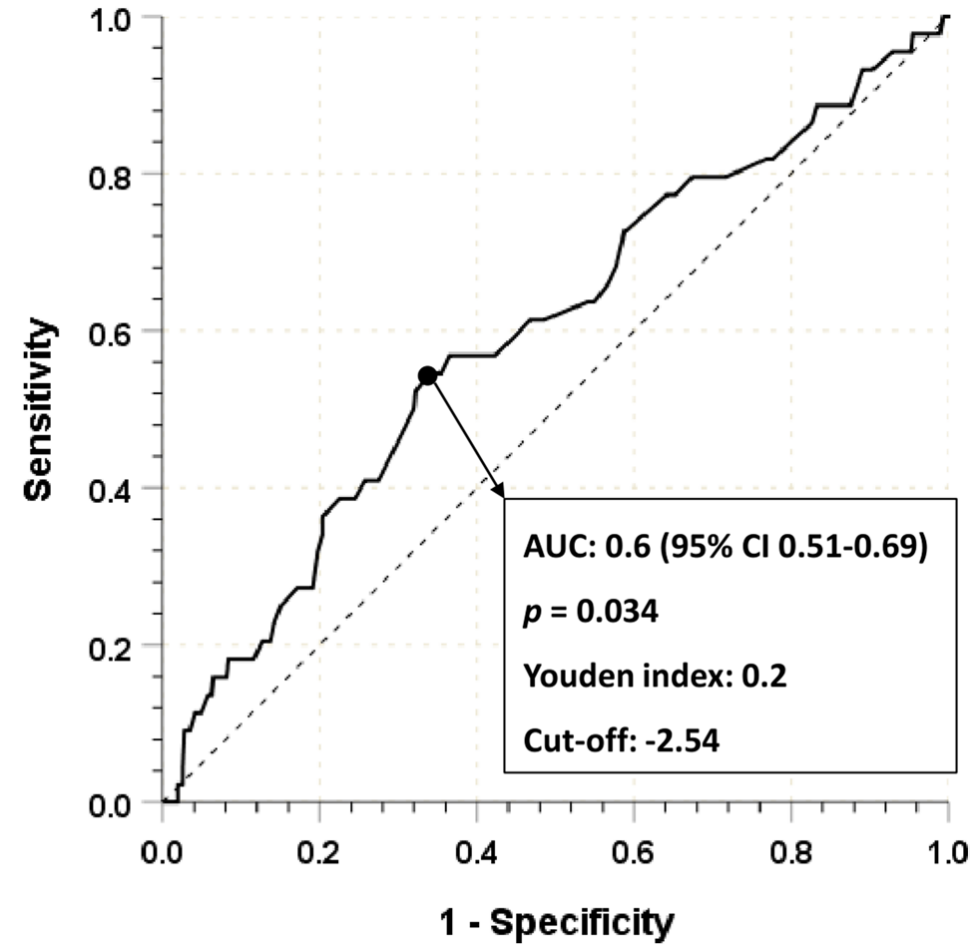
ROC curve for cancer-specific survival to determine an optimal cut-off value for the ALBI score.

Table 1 shows the clinicopathological characteristics of the 510 patients who participated in the study, and the median follow-up period was 59 months. The average ALBI score was − 2.68 (range from − 3.39 to − 0.69). Using ROC curves and the Youden Index, the optimal cut-off value was − 2.54 (AUC = 0.6, 95% CI 0.51–0.69, *p* = 0.034, Youden Index = 0.2, Fig. 1) and patients were divided into two groups: Group A (ALBI score ≤  − 2.54) and Group B (ALBI score >  − 2.54). The mean serum albumin levels in Group A were 3.9 ± 0.3 g/dL and 3.3 ± 0.4 g/dL in Group B (*p* < 0.001). Patients in Group B were older than those in Group A, had a lower BMI, and had higher ASA scores. Group B had a higher proportion of obstruction and a more advanced pathologic T category. After adjusting these differences between two ALBI groups by PSM, there was no significant between-group differences except for ASA score and surgical approach. The patient number in Groups A and B after PSM were 173, respectively. Before PSM, 93.0% of the patients in Group A received adjuvant chemotherapy and 5.9% did not complete their recommended chemotherapy cycle, whereas 80.2% of patients in Group B received adjuvant chemotherapy and 13.2% of them discontinued chemotherapy. There was no significant between-group difference in the proportion of patients treated with adjuvant chemotherapy after PSM, however, chemotherapy discontinuation rate in Group A was significantly lower than that in Group B.

**Table 1:** Clinicopathologic characteristics according to albumin–bilirubin (ALBI) score.

Characteristic	Before PSM^a^			After PSM^a^		
	Group A (n = 328)	Group B (n = 182)	*P*-value^b^	Group A (n = 173)	Group B (n = 173)	*P*-value^b^
**Sex**			0.98			0.389
Male	177 (54.0)	98 (53.8)		88 (50.9)	96 (55.5)	
Female	151 (46.0)	84 (46.2)		85 (49.1)	77 (44.5)	
Age, years	58.5 ± 12.1	64.8 ± 11.9	< 0.001	63.3 ± 10.6	64.0 ± 11.7	0.581
BMI, kg/m^2^	23.9 ± 3.2	23.4 ± 3.1	0.084	23.4 ± 3.0	23.5 ± 3.1	0.818
Albumin level, g/dL	3.9 ± 0.3	3.3 ± 0.4	< 0.001	3.9 ± 0.3	3.3 ± 0.4	< 0.001
Bilirubin level, mg/dL	0.4 ± 0.3	0.5 ± 0.3	0.01	0.4 ± 0.2	0.5 ± 0.3	0.003
ASA score III–IV	7 (2.1)	17 (9.3)	< 0.001	6 (3.5)	15 (8.7)	0.043
History of liver disease	5 (1.5)	5 (2.7)	0.34	4 (2.3)	5 (2.9)	0.736
CEA > 5 ng/ml	68 (20.7)	50 (27.5)	0.084	37 (21.4)	47 (27.2)	0.21
Obstruction	21 (6.4)	26 (14.3)	0.003	15 (8.7)	23 (13.3)	0.169
**Surgical approach**			< 0.001			0.046
Open	63 (19.2)	61 (33.5)		43 (24.9)	60 (34.7)	
Laparoscopic/robotic	265 (80.8)	121 (66.5)		130 (75.1)	113 (65.3)	
**pT category**			0.001			0.666
T1–2	65 (19.8)	14(7.7)		17 (9.8)	14 (8.1)	
T3	195 (59.5)	128 (70.3)		122 (70.5)	119 (68.8)	
T4	68 (20.7)	40 (22.0)		34 (19.7)	40 (23.1)	
**pN category**			0.811			0.804
N1	250 (76.2)	137 (75.3)		131 (75.7)	129 (74.6)	
N2	78 (23.8)	45 (24.7)		42 (24.3)	44 (25.4)	
**Differentiation**			0.162			0.311
WD + MD	296 (90.8)	158 (86.8)		156 (90.7)	151 (87.3)	
PD + Muc + SRC	30 (9.2)	24 (13.2)		16 (9.3)	22 (12.7)	
**Adjuvant chemotherapy**			< 0.001			0.179
No	14 (4.3)	29 (15.9)		12 (6.9)	22 (12.7)	
Yes	305 (93.0)	146 (80.2)		153 (88.4)	145 (83.8)	
Unknown	9 (2.7)	7 (3.8)		8 (4.6)	6 (3.5)	
**Chemotherapy regimen**			0.206			0.374
FOLFOX	133 (43.5)	66 (44.9)		63 (40.9)	66 (45.2)	
CAPOX	128 (41.8)	50 (34.0)		61 (39.6)	49 (33.6)	
Capecitabine	25 (8.2)	13 (8.8)		19 (12.3)	13 (8.9)	
LV/5FU	7 (2.3)	8 (5.4)		6 (3.9)	8 (5.5)	
Unknown	13 (4.2)	10 (6.8)		5 (3.2)	10 (6.8)	
Chemotherapy discontinuation	17 (5.9)	18 (13.2)	0.011	9 (6.1)	18 (13.3)	0.04

*PSM* propensity score-matching, *ALBI* albumin–bilirubin, *BMI* body mass index, *ASA* American Society of Anesthesiologists Classification, *CEA* preoperative carcinoembryonic antigen, *WD* well differentiated, *MD* moderately differentiated, *PD* poorly differentiated, *Muc* mucinous carcinoma, *SRC* signet ring cell carcinoma, *FOLFOX* leucovorin, fluorouracil, and oxaliplatin, *CAPOX* capecitabine and oxaliplatin, *LV/5FU* leucovorin and fluorouracil.

^a^Sex, age, BMI, and pT category were adjusted by propensity score-matching.

^b^Categorical variables were compared by Fisher's exact test or Pearson's chi-squared test, as appropriate; continuous variables were compared by unpaired *t* tests.

### Postoperative complications

There were 37 (10.7%) postoperative complications among the PSM cohorts, with 16 (4.6%) experiencing severe complications (Table 2). Postoperative ileus was the most common complication (16 patients, 4.6%), accounting for 40% of all complications. The rate of postoperative complications was 9.8% in Group A and 11.6% in Group B, with no statistically significant difference. The rates of severe complications did not differ between Groups A and B.

**Table 2:** Postoperative complications according to the ALBI score.

	ALBI score			
	Total, n = 346	Group A, n = 173	Group B, n = 173	*P*-value^a^
Postoperative complications	37 (10.7)	17 (9.8)	20 (11.6)	0.602
Anastomotic leakage	4 (1.2)	1 (0.6)	3 (1.7)	0.314
Ileus	16 (4.6)	9 (5.2)	7 (4.0)	0.609
Wound infection	3 (0.9)	1 (0.6)	2 (1.2)	0.562
Bleeding	2 (0.6)	1 (0.6)	1 (0.6)	1
Incisional hernia	7 (2.0)	3 (1.7)	4 (2.3)	0.703
Other	6 (1.7)	3 (1.7)	3 (1.7)	1
Severe complications^b^ (≥ Dindo IIIb)	16 (4.6)	9 (5.2)	7 (4.0)	0.609

^a^Compared by Fisher's exact test or Pearson's chi-squared test.

^b^Severe grade was defined as Clavien–Dindo grade IIIb or higher.

### Prognostic impact of ALBI score

**Figure 2:**
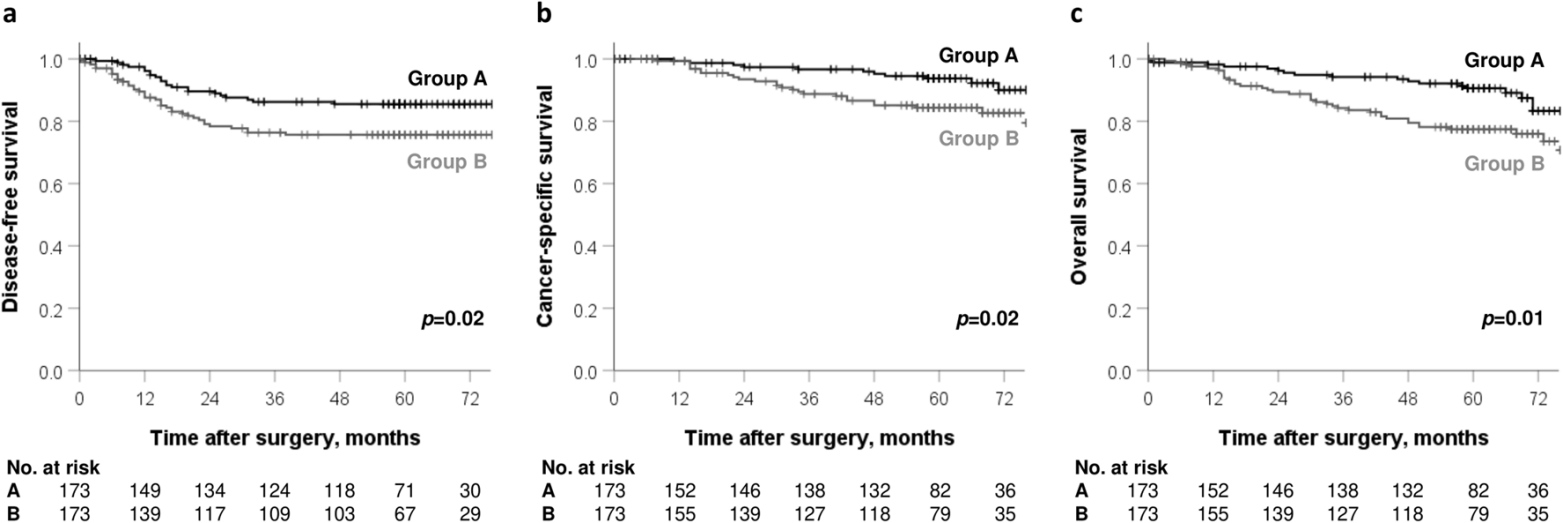
Survival analysis in Group A and Group B. (**a**) Cumulative 5-year disease-free survival (DFS), (**b**) cancer-specific survival (CSS) and (**c**) overall survival (OS).

**Figure 3:**
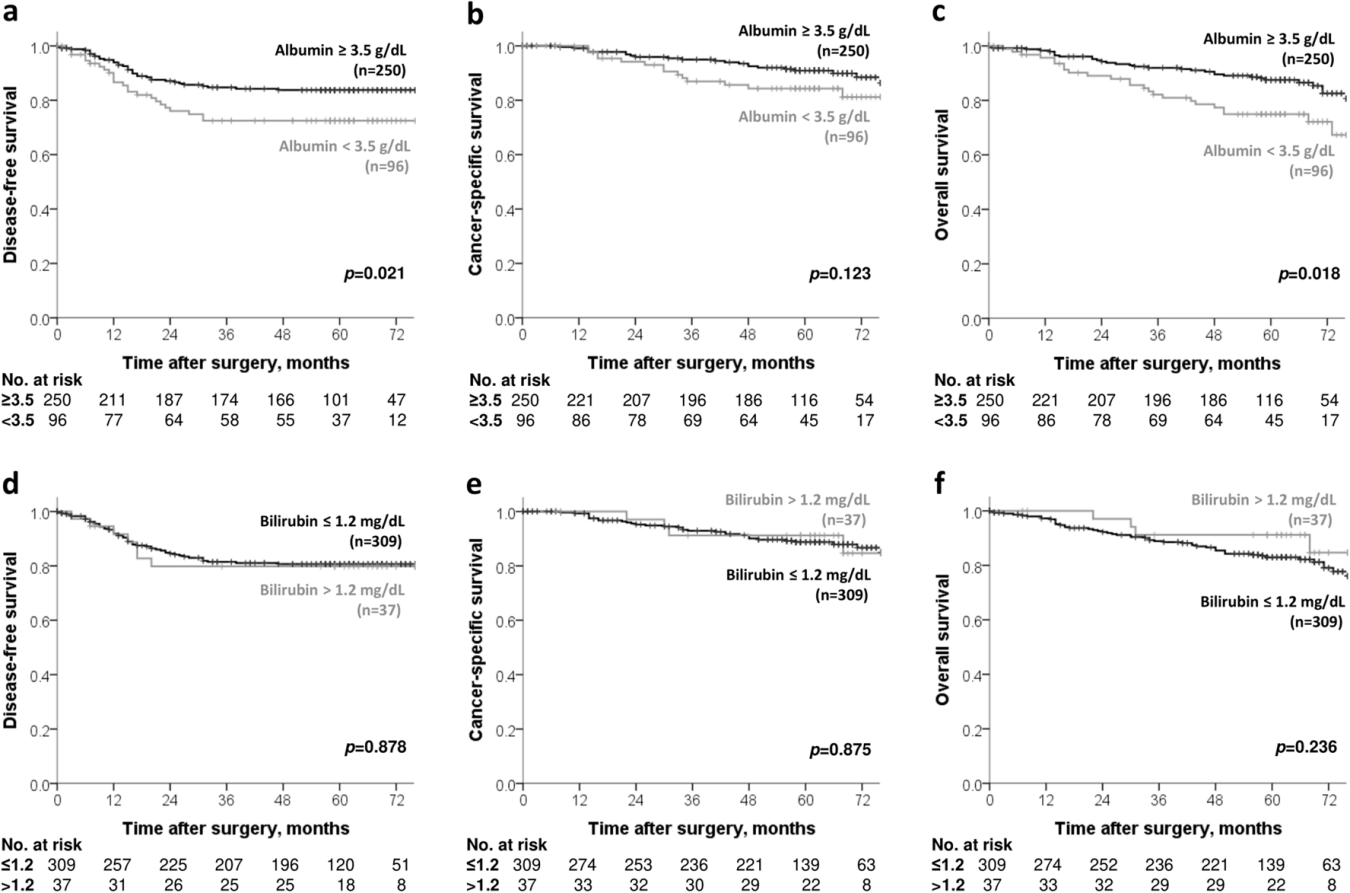
Survival analysis according to serum albumin and bilirubin levels. (**a**) Cumulative 5-year DFS, (**b**) CSS, and (**c**) OS according to serum albumin level. (**d**) Cumulative 5-year DFS, (**e**) CSS, and (**f**) OS according to serum bilirubin level.

The 5-year DFS rate in Group A was higher than in Group B (85.5% vs 75.7%, *p* = 0.02, Fig. 2a). The 5-year CSS and OS rates in Group A were also significantly higher those in Group B (CSS 93.7% vs 84.4%, p = 0.02, OS 90.6% vs 77.4%, *p* = 0.01, Fig. 2b,c). Serum albumin and bilirubin levels were evaluated separately for prognosis (Fig. 3), and patients with low serum albumin levels (< 3.5 g/dL) had significantly shorter DFS and OS than those with normal serum albumin levels.

In univariate analysis, a high-ALBI score was associated with DFS along with a low serum albumin level, pathologic T category, and pathologic N category (Table 3). High ALBI score (HR 1.68, *p* = 0.048), and pathologic T4 (HR 5.41, *p* = 0.024) were identified as independent risk factors for DFS in multivariate analysis. A high ALBI score was also associated with CSS and OS independently of age > 65 and pathologic N2 (Table 4).

**Table 3:** Clinicopathological factors associated with disease-free survival, cancer-specific survival, and overall survival in univariate analysis.

Factors	DFS		CSS		OS	
	HR (95% CI)	*P*-value^a^	HR (95% CI)	*P*-value^a^	HR (95% CI)	*P*-value^a^
Sex (female/male)	0.86 (0.51–1.43)	0.557	0.76 (0.39–1.48)	0.425	1.25 (0.75–2.07)	0.388
Age (> / ≤ 65 years)	1.47 (0.89–2.44)	0.137	2.33 (1.2–4.45)	0.012*	4.09 (2.32–7.19)	< 0.001*
**BMI**						
Normal (18.5–23)	1		1		1	
Underweight (< 18.5)	1.05 (0.32–3.45)	0.941	1.94 (0.57–6.62)	0.293	2.36 (0.8–6.9)	0.119
Overweight (23–25)	0.61 (0.31–1.21)	0.155	0.42 (0.16–1.14)	0.088	1.07 (0.54–2.14)	0.848
Obese (≥ 25)	0.83 (0.46–1.5)	0.53	0.92 (0.44–1.92)	0.82	1.61 (0.88–2.97)	0.124
ASA score (III–IV/I–II)	1.02 (0.32–3.27)	0.968	1.18 (0.28–4.9)	0.824	2.52 (1.14–5.56)	0.023*
Alb, g/dL (< / ≥ 3.5)	1.82 (1.09–3.05)	0.023*	1.68 (0.86–3.26)	0.128	1.85 (1.1–3.09)	0.02*
Bil, mg/dL (> / ≤ 1.2)	0.96 (0.57–1.61)	0.872	1.05 (0.54–2.02)	0.894	1.27 (0.77–2.12)	0.354
ALBI score (high/low)	1.85 (1.09–3.12)	0.022*	2.21 (1.11–4.4)	0.024*	1.98 (1.16–3.37)	0.012*
CEA, ng/ml (> / ≤ 5)	1.58 (0.92–2.7)	0.095	1.58 (0.79–3.15)	0.194	1.43 (0.82–2.49)	0.205
Obstruction (yes/no)	1.34 (0.63–2.81)	0.447	2.04 (0.89–4.64)	0.091	1.53 (0.75–3.1)	0.242
Approach (Open/MIS)	0.7 (0.41–1.18)	0.181	0.78 (0.4–1.5)	0.469	0.7 (0.42–1.18)	0.182
**pT category**						
T1–2	1		1		1	
T3	2.22 (0.53–9.26)	0.276	1.89 (0.61–6.21)	0.321	1.65 (0.51–5.37)	0.407
T4	6.88 (1.64–28.94)	0.009*	5.72 (1.42–20.6)	0.023*	4.39 (1.31–14.7)	0.016*
pN category (N2/N1)	1.76 (1.03–3.01)	0.04*	2.43 (1.26–4.7)	0.008*	2.15 (1.26–3.65)	0.005*
**Differentiation**						
WD + MD	1		1		1	
PD + Muc + SRC	2.15 (1.12–4.15)	0.022*	3.61 (1.68–7.74)	0.001*	2.56 (1.32–4.96)	0.006*

*DFS* disease-free survival, *CSS* cancer-specific survival, *OS* overall survival, *HR* hazard ratio, *ALBI* albumin–bilirubin, *Alb* serum albumin level, *Bil* serum bilirubin level, *CEA* preoperative carcinoembryonic antigen, *MIS* minimally invasive surgery (laparoscopic and robotic), *WD* well differentiated, *MD* moderately differentiated, *PD* poorly differentiated, *Muc* mucinous carcinoma, *SRC* signet ring cell carcinoma.

*P-value < 0.05.

^a^Significant variables were identified by univariate analysis using Cox proportional hazard regression model.

**Table 4:** Clinicopathological factors associated with disease-free survival, cancer-specific survival, and overall survival in multivariate analysis.

Factors	DFS		CSS		OS	
	HR (95% CI)	*P*-value^a^	HR (95% CI)	*P*-value^a^	HR (95% CI)	*P*-value^a^
**Sex (female/male)**						
Age (> / ≤ 65 years)			2.1 (1.06–4.17)	0.033*	4.06 (2.25–7.32)	< 0.001*
**BMI**						
Normal (18.5–23)						
Underweight (< 18.5)						
Overweight (23–25)						
Obese (≥ 25)						
ASA score (III–IV/I–II)					2.29 (0.97–5.4)	0.059
Alb, g/dL (< / ≥ 3.5)	1.02 (0.53–1.96)	0.947			0.8 (0.41–1.56)	0.512
**Bil, mg/dL (> / ≤ 1.2)**						
ALBI score (high/low)	1.68 (1.01–2.78)	0.048*	2.24 (1.09–4.56)	0.028*	2 (1.05–3.84)	0.036*
CEA, ng/ml (> / ≤ 5)						
Obstruction (yes/no)						
Approach (Open/MIS)						
**pT category**						
T1–2	1		1		1	
T3	1.93 (0.46–8.2)	0.371	1.56 (0.55–5.33)	0.425	1.405 (0.42–4.7)	0.581
T4	5.41 (1.24–23.58)	0.024*	4.28 (1.23–15.6)	0.09	3.32 (0.94–11.8)	0.063
pN category (N2/N1)	1.71 (0.99–2.94)	0.054	2.24 (1.13–4.43)	0.021*	2.12 (1.23–3.66)	0.007*
**Differentiation**						
WD + MD	1		1		1	
PD + Muc + SRC	1.52 (0.77–2.99)	0.233	2.03 (0.92–4.51)	0.082	1.73 (0.86–3.47)	0.124

*DFS* disease-free survival, *CSS* cancer-specific survival, *OS* overall survival, *HR* hazard ratio, *ALBI* albumin–bilirubin, *Alb* serum albumin level, *Bil* serum bilirubin level, *CEA* preoperative carcinoembryonic antigen, *MIS* minimally invasive surgery (laparoscopic and robotic), *WD* well differentiated, *MD* moderately differentiated, *PD* poorly differentiated, *Muc* mucinous carcinoma, *SRC* signet ring cell carcinoma.

*P-value < 0.05.

^a^Significant variables were identified by univariate analysis using Cox proportional hazard regression model.

## Discussion

The ALBI score has been validated as a useful predictor of hepatic dysfunction using only two variables, serum albumin, and bilirubin^10,11^. We discovered that the preoperative ALBI score had a significant association with recurrence and survival in this study of patients with stage III colon cancer who underwent radical resection followed by adjuvant chemotherapy. Postoperative complication rates, on the other hand, did not differ between Groups A and B.

Our study found that Group A had significantly higher DFS, CSS, and OS rates than Group B. Furthermore, a high ALBI score was found to be an independent risk factor for DFS, CSS, and OS. These findings are consistent with those of a previous study that found a link between the ALBI score and survival in patients with colorectal cancer who did not have distant metastasis ^17^. Although that previous study included patients with stage I-III colorectal cancer, the ALBI score was only a significant prognostic factor in stage III patients. It also had the limitation of a small sample size (n = 284) and was unable to assess the relationship between the ALBI score and chemotherapy. As a result, our study focused on patients with stage III disease, and we investigated the relationship between the ALBI score and adjuvant chemotherapy. Although adjuvant chemotherapy has been the standard of care for patients with stage III colon cancer, 13.3% of patients in Group B did not receive it in this study. Furthermore, Group A had a significantly higher chemotherapy completion rate than Group B even after adjusting baseline characteristics such as age. These findings are consistent with previous research, which found that the high-ALBI group had a higher proportion of patients who discontinued chemotherapy than the low-ALBI group^16^. Because the liver is involved in drug metabolism, patients with liver dysfunction are more susceptible to the toxic effects of chemotherapy agents^22^. Impairment of liver metabolic and excretory function, as indicated by a high ALBI score, may have resulted in chemotherapy-induced side effects, resulting in early discontinuation of chemotherapy. This could be one of the mechanisms by which the ALBI score can be used as an independent prognostic factor in patients with stage III colon cancer.

Previous studies^9,11^ used cut-off values of ALBI grades of − 2.60 and − 1.39, which were not appropriate for colon cancer patients because the majority of them had a normal hepatic function and only a few patients had a history of liver disease. In our dataset, the optimal cut-off value was − 2.54, and the groups divided according to this cut-off value showed significant differences in survival outcomes. Despite statistical differences in albumin and bilirubin levels between the two groups, serum albumin levels may have a greater impact on prognosis. Albumin is a protein that is specifically synthesized in the liver, and serum albumin levels are commonly used to assess liver synthetic function and nutritional status^23^. Nutrition has been shown to be closely related to the immune system. Impaired nutritional status can suppress the anticancer immune response, causing tumor progression to be accelerated^24,25^. Patients with low serum albumin levels had shorter DFS and OS in our study, which can be attributed to the effects of impaired nutritional status as reflected by serum albumin levels. However, in the multivariate analysis, serum albumin level alone was not a significant prognostic factor, whereas ALBI score, when combined with serum bilirubin level, was an independent factor for recurrence and survival.

Bilirubin has not yet been clearly associated with the prognosis of colon cancer^26^, and there was no difference in oncologic outcome according to bilirubin level in this study. It is reported that liver-derived metabolites such as bilirubin may affect the composition of the gut microbiota and have a significant role in gut homeostasis and host defense^27^. It is also reported that change in the gut microbiota plays an important role in the cancer microenvironment affecting the development and recurrence of colon cancer. In particular, a decrease in *B. vulgatus* and increase in *P. mirabilis* was related to a decrease in Kupfer cells in the liver, which was linked to liver metastasis^28^. Further studies on the relationship between gut homeostasis and the molecules such as bilirubin excreted from the liver may help explain the association between ALBI score and colon cancer prognosis.

Chronic liver disease has been linked to an increased risk of morbidity and mortality following colorectal cancer surgery^4^. Although Group A had fewer postoperative complications than Group B, the difference was not statistically significant. According to recent research, a high ALBI score is associated with a higher risk of medical and severe complications, but not surgical complications^17^. Although the classification of postoperative complications was different, the finding that there was no significant difference in surgical complications based on the ALBI score was similar to the findings of our study. The frequency of postoperative complications has gradually decreased as surgical techniques have advanced and the proportion of minimally invasive approaches has increased. Furthermore, this study only included patients with colon cancer, and the complication rate of colon cancer surgery is lower than that of rectal cancer surgery. As a result of these factors, the occurrence of complications was relatively low, and the ALBI score may have had little effect on postoperative complications. Large-scale studies should be conducted in the future to confirm the correlation between the ALBI score and postoperative complications.

There were several limitations to this study. First, the findings of this were derived from retrospective data from a tertiary specialized center. Although PSM analysis was performed to adjust differences in ALBI groups, this study still has selection bias. Second, the applicability of this prognostic index derived from a single center can be limited without sufficient validation. Further research in a multicenter setting should be conducted to validate the findings of this study. Third, only patients with stage III colon cancer were analyzed in this study, so the prognostic impact of the ALBI score on stage I–II or metastatic disease should be confirmed by future studies. Fourth, the mechanism by which the ALBI score affects colon cancer prognosis remains unknown and will need to be explained in future studies.

In conclusion, the ALBI score appears to be a promising prognostic biomarker for predicting recurrence and survival in patients with stage III colon cancer. The ALBI score has the potential to be a useful clinical marker for assessing the prognosis of patients with colon cancer because it can be obtained easily, less invasively, and at a low cost. Future research on tailored treatment strategies will be required to improve the prognosis of patients with high ALBI scores.

## Data Availability

All data generated or analysed during this study are included in this published article.
